# “Have You Ever Seen This Face?” – Individual Differences and Event-Related Potentials during Deception

**DOI:** 10.3389/fpsyg.2012.00570

**Published:** 2012-12-20

**Authors:** Anja Leue, Sebastian Lange, André Beauducel

**Affiliations:** ^1^Clinic of Epileptology, University of BonnBonn, Germany; ^2^Institute of Psychology, University of BonnBonn, Germany

**Keywords:** deception, P3, MFN, individual differences, fixed-links modeling

## Abstract

Deception studies emphasize on the importance of event-related potentials (ERP) for a reliable differentiation of the underlying neuro-cognitive processes. The stimulus-locked parietal P3 amplitude has been shown to reflect stimulus salience but also attentional control available for stimulus processing. Known stimuli requiring truthful responses (targets) and known stimuli requiring deceptive responses (probes) were hypothesized to be more salient than unknown stimuli. Thus, a larger P3 was predicted for known truthful and deceptive stimuli than for unknown stimuli. The Medial Frontal Negativity (MFN) represents the amount of required cognitive control and was expected to be more negative to known truthful and deceptive stimuli than to unknown stimuli. Moreover, we expected higher sensitivity to injustice (SI-perpetrator) and aversiveness (Trait-BIS) to result in more intense neural processes during deception. *N* = 102 participants performed a deception task with three picture types: probes requiring deceptive responses, targets requiring truthful responses to known stimuli, and irrelevants being associated with truthful responses to unknown stimuli. Repeated-measures ANOVA and fixed-links modeling suggested a more positive parietal P3 and a more negative frontal MFN to deceptive vs. irrelevant stimuli. Trait-BIS and SI-perpetrator predicted an increase of the P3 and a decrease of the MFN from irrelevants to probes. This suggested an intensification of stimulus salience and cognitive control across picture types in individuals scoring either higher on Trait-BIS or higher on SI-perpetrator. In contrast, individuals with both higher Trait-BIS and higher SI-perpetrator scores showed a less negative probe-MFN suggesting that this subgroup invests less cognitive control to probes. By extending prior research we demonstrate that personality modulates stimulus salience and control processes during deception.

## Introduction

One of the main interests in forensic psychophysiology refers to the differentiation of truthful and deceptive responses. Referring to different cognitive models on deception (e.g., Zuckerman et al., 1981; Walczyk et al., 2003), there is a considerable number of studies investigating the underlying processes of deception by means of verbal and non-verbal behavior (DePaulo et al., 2003) or behavioral parameters (Zuckerman et al., 1981). Moreover, the relevance of the P3 amplitude of the event-related potential (ERP) has been originally demonstrated for persons who recognize items on that information should be concealed (sometimes named as guilty group) vs. persons who do not recognize those items (sometimes named as innocent group) because they are unfamiliar with (Rosenfeld et al., 1988; Farwell and Donchin, 1991). These studies encouraged a growing research interest in the modulation of ERPs in deception settings and in elucidating the underlying neuro-cognitive processes of deceptive vs. truthful responses. The relevance of ERPs for the differentiation of deceptive vs. truthful responses has been successfully illustrated in guilty knowledge tasks (GKT, also named as concealed information test, CIT) and other deception tasks (Farwell and Donchin, 1991; Allen et al., 1992; Fang et al., 2003; Johnson et al., 2008; Ambach et al., 2010; Gamer and Berti, 2010).

A considerable number of studies investigated variations of the P3 component for deceptive vs. truthful stimuli by means of CITs (e.g., Rosenfeld et al., 1988, 1991; Farwell and Donchin, 1991; Mertens and Allen, 2008; Ambach et al., 2010; Gamer and Berti, 2010; Meixner and Rosenfeld, 2011) or visual recognition tasks (Fang et al., 2003; Meijer et al., 2007; Dong et al., 2010a). In deception tasks, participants learn subsets of stimuli that require deceptive vs. truthful responses prior to task performance. Most P300-based CITs comprise three types of stimuli: probe, target, and irrelevant stimuli. Probe stimuli are deception-relevant stimuli that are known by participants who are requested to deceive the knowledge of these stimuli in their responses. Target stimuli are known by participants and they require truthful responses. Target stimuli are useful to ensure that participants attend to the presented stimuli and do not ignore them (cf. Farwell and Donchin, 1991; Fang et al., 2003; Mertens and Allen, 2008; Gamer and Berti, 2010). Irrelevant stimuli incorporate stimuli that participants have not seen before task performance (e.g., Meijer et al., 2009; Meixner and Rosenfeld, 2011). All deception tasks have in common that individuals “need to consciously select and execute a response that is incompatible with the truth…” (Johnson et al., 2008, p. 469). Accordingly, executive processes like attentional control and cognitive control should play an important role during deception (e.g., Gombos, 2006; Carrión et al., 2010).

Although different conceptual meanings have been discussed for the P3 amplitude depending on tasks and context (Mulder, 1986; Mecklinger et al., 1992; Kok, 2001; Beauducel et al., 2006; Polich, 2007), the parietal P3 amplitude (mainly occurring between 300 and 800 ms post-stimulus) is one of the most frequently investigated ERPs in deception tasks. In some studies on P3 and deception, the P3 is regarded as an indicator of task relevance or stimulus salience leading to larger P3 amplitudes for target and probe stimuli compared to irrelevant stimuli (e.g., Ambach et al., 2010; Gamer and Berti, 2010). Moreover, deceptive responses might involve additional processes related to justification or ethical discomfort that are not relevant for target stimuli. Moreover, effects of personality on P3 amplitudes have been demonstrated in different quasi-experimental settings (Beauducel et al., 2006; Leue et al., 2009; Wacker et al., 2010). The P3 amplitude captures individual differences that have been related to stimulus salience or stimulus complexity (Stenberg, 1994; Fink and Neubauer, 2004) and cognitive resources (Beauducel et al., 2006).

In addition to P3-related processes of salience and attentional control, response-related cognitive control has been discussed as a neuro-cognitive process during deception because individuals either have to adapt their responses to deceptive information in comparison to truthful information or they have to inhibit responses in order to successfully conceal knowledge. In this respect, the response-locked Medial Frontal Negativity (MFN) has been investigated as an indicator of cognitive control (e.g., Johnson et al., 2004, 2008). The MFN has a fronto-central topography and occurs 0–70 ms post-response in deception settings (Johnson et al., 2008). In non-deception settings, the MFN (or Error Related Negativity) is more negative when actions fail to meet motivational goals (Potts et al., 2006) and following erroneous responses compared to correct responses (e.g., Luu et al., 2000). Presuming that individuals interpret deceptive responses as erroneous responses (i.e., violating social norms), deceptive responses should be more aversive than truthful responses. Therefore, the MFN should be more negative following deceptive responses than following truthful responses (cf. Dong et al., 2010a, 2011).

To the best of our knowledge, there is no P3-study relating personality traits and neural responses to deceptive vs. truthful stimuli, whereas trait-related differences of the P3 have been intensely studied in other contexts (Stenberg, 1994; Fink and Neubauer, 2004; Beauducel et al., 2006; Leue et al., 2009; Wacker et al., 2010). However, studies on cognitive control reported that personality dimensions like fairness concerns modulate the feedback-locked MFN amplitude (Boksem and De Cremer, 2010). More precisely, Boksem and De Cremer (2010) reported a more negative MFN in an ultimatum game for individuals with high compared to low scores in moral identity. A conflict based on an individuals’ moral and social standards could also be induced by the instruction to conceal information. Therefore, individuals who are highly sensitive to moral and social norms should demonstrate a more negative MFN to probe compared to target and irrelevant items in a deception task. A personality dimension that might correspond to an individuals’ sensitivity to moral and social norms is the trait-dimension sensitivity to injustice (SI, Schmitt et al., 2005). Another trait-dimension that reflects personality differences of cognitive-motivational conflict processing is Trait-BIS (Carver and White, 1994). Trait-BIS refers to the activation of the behavioral inhibition system (BIS) that serves as a device for conflict detection and resolution (Gray and McNaughton, 2000; Corr, 2008). Individuals with higher Trait-BIS scores show a more pronounced BIS activation compared to lower Trait-BIS individuals. Trait-BIS has been shown to reflect aversiveness sensitivity in conflict and non-conflict situations (Leue and Beauducel, 2008). Because deception might induce conflict with one’s social and moral standards (to respond honestly) deception could be aversive especially to higher vs. lower Trait-BIS individuals. This enhanced sensitivity to aversiveness might increase the salience of probes for higher vs. lower Trait-BIS individuals. Accordingly, we investigated whether probe stimuli compared to target and irrelevant stimuli are more salient for higher vs. lower Trait-BIS individuals. If this prediction would be true, a larger P3 amplitude should be observed in higher vs. lower Trait-BIS individuals for probe stimuli compared to target and irrelevant stimuli. Probe stimuli compared to target and irrelevant stimuli might be of special salience to higher Trait-BIS individuals because lying is an aversive event, and higher Trait-BIS individuals should be more sensitive to aversive events (cf. Corr, 2008; Leue and Beauducel, 2008). Moreover, according to the above-cited literature we hypothesized a more intense cognitive control of higher Trait-BIS individuals for probe stimuli relative to target and irrelevant stimuli. This should be indicated by a more negative probe-MFN in higher Trait-BIS individuals compared to lower Trait-BIS individuals. We also aimed at probing whether individuals with a higher sensitivity to social norms (i.e., higher SI scores) and a higher sensitivity to aversiveness (i.e., Trait-BIS) show more pronounced P3 and MFN-amplitudes on deceptive stimuli compared to truthful stimuli. Altogether, the present study aimed at investigating neuro-cognitive processes of deception – namely stimulus salience or attentional control (by means of stimulus-locked P3) and cognitive control (by means of response-locked MFN) – as well as the modulation of these processes by individual differences of SI and Trait-BIS.

From a more general point of view the investigation of inter-individual differences within deception processes brings together the correlative personality research tradition with the tradition of experimental deception research. The crossbreeding of correlative and quasi-experimental research has been promoted intensely since Cronbach (1975) so that the methodological approaches for modeling individual differences within repeated-measures designs have also been improved (Raudenbush and Bryk, 2002; Muthén, 2004). For example, Raudenbush and Bryk (2002) described individual differences as random effects in the context of hierarchical linear models whereas Muthén (2004) proposed a modeling of the individual differences together with treatment effects as latent variables in the context of structural equation modeling (SEM). Both approaches have their merits, but in the present context the modeling of individual differences and treatment effects as latent variables was considered as an advantage, because latent variables are regarded as more appropriate indicators of psychological constructs than measured variables. Thus, measurement models can be specified in SEM that allow for a separation of construct relevant common variance represented by latent variables from the specific error variance. Since this separation is only possible with SEM, this framework was regarded as most appropriate for the present study (see Materials and Methods for further specifications). Nevertheless, conventional analyses of variance (ANOVA) were also reported in order to facilitate comparisons with previous research. Altogether, the complex aim of investigating individual differences (i.e., Trait-BIS, SI) in conjunction with treatment effects in a deception task fits well to the flexibility of the SEM approach.

## Materials and Methods

### Participants

A total of *N* = 114 students from a German University participated individually in the present study. Written informed consent has been given by all participants. Artifacts that could not be corrected by means of Independent Component Analysis (ICA; see below) resulted in an in-sufficient number of trials per picture type (i.e., less than 20 trials per picture type) in 12 participants so that a sample of *N* = 102 (48 male, age: *M* = 23.80 years, SD = 3.75, range: 19–37 years) participants remained for the analysis of the P3 components. Due to an increased number of muscle artifacts during response preparation, a sample of *N* = 91 participants (42 male) was available for the analysis of the MFN. Based on the Edinburgh Handedness Inventory (Oldfield, 1971) all included participants were right-handed. The ethical standards of this study were approved by the ethical commission of the German Research Foundation.

### Measures

Participants filled in the German version of the BIS/BAS scales (Strobel et al., 2001). The BIS/BAS scales measure an individual’s sensitivity to aversiveness (Trait-BIS) and an individuals’ sensitivity to appetitive reinforcement (Trait-BAS) with 24-items using a five-point Likert-type answer format. The Trait-BIS scale is an established personality scale in studies investigating individual differences of cognitive control (e.g., Boksem et al., 2006; Amodio et al., 2008; Lange et al., 2012; Leue et al., 2012a,b). Therefore, the Trait-BIS scale was applied to investigate individual differences of the P3 and the MFN in our deception study (Cronbach’s α: 0.80). The Sensitivity to Injustice questionnaire (Schmitt et al., 2005) measures individual differences of SI for different perspectives (perpetrator, victim, observer, and one’s favor) and consists of 40 items with a seven-point answer format (0 = not at all, 6 = strong agreement). To elucidate those individual differences of deception that might be related to justice or fairness concerns, we focused on the SI-perpetrator subscale (10 items) in our ERP analyses (Cronbach’s α: 0.87) because this subscale is related to an individual’s moral standards of feeling guilty when he/she treats others unfairly. Trait-BIS and SI-perpetrator correlated significantly, *r*(102) = 0.24, *p* < 0.05 (two-tailed).

### Deception task

The present task incorporated three types of pictures that were taken from the International Affective Picture System (IAPS, Bradley and Lang, 2007) and the task was designed in accordance with the study of Fang et al. (2003). All selected pictures showed either a face of a woman or a face of a man. Regarding “probe” pictures (number of IAPS pictures: 2190, 2516, 2214), participants were asked to conceal their knowledge by pressing on the left cursor button as required for the irrelevant pictures (see below). On “target” pictures (number of IAPS pictures: 2500, 2305, 2215), participants should indicate truthfully by a button press on the right cursor button that they knew the pictures. Finally, there was a total of 20 “irrelevant” pictures that were completely unknown to the participants. Participants were asked to indicate truthfully by pressing on the left cursor button that they did not know them (number of IAPS pictures: 2372, 2383, 2512, 2200, 2210, 2221, 2630, 2104, 2102, 2495, 2510, 2230, 2005, 2020, 2493, 2000, 2010, 2385, 2499, 2513). We chose a large number of different pictures that participants have not seen before performing the task to ensure that these irrelevant pictures would remain rather strange and, thus, of low relevance throughout the experimental task. Averaged valence and arousal values have been calculated based on the IAPS manual for probe pictures, target pictures, and irrelevant pictures. Means of the valence dimension were widely comparable for the three picture types (probe: *M* = 4.91, SD = 0.09; target: *M* = 5.40, SD = 0.77, irrelevant: *M* = 5.34, SD = 0.76). The same was true for means of the arousal dimension (probe: *M* = 3.12, SD = 0.62; target: *M* = 3.54, SD = 0.14; irrelevant: *M* = 3.48, SD = 0.37).

All task-related instructions were presented on the screen. To make the requirement of concealing knowledge more salient, participants were encouraged to give their best so that the computer program could not detect based on EEG and response data when participants concealed knowledge to the probe pictures. In accordance with Fang et al. (2003), participants received the information that if the computer program recognized deception, they would lose 15 Cent even if they pressed the correct cursor button. Otherwise they would win 5 Cent. Altogether participants performed 150 trials (50 probe, 50 irrelevant, and 50 target items) presented in a pseudo-random order with a 2-min break after 75 trials. In order to realize the traditionally applied ratio with less frequently occurring probe and target stimuli relative to irrelevant stimuli, three different probe and three different target pictures were selected, whereas 20 different irrelevant pictures were chosen. Thus, the number of three different probe, three different irrelevant pictures, and three different target pictures followed a 3:20:3 ratio, which is comparable to other studies (e.g., Meijer et al., 2007). Thus, per task block (including 75 trials) each of the three probe pictures and each of the three target pictures was presented about eight times and most of the 20 irrelevant pictures were applied once.

**Figure 1:**
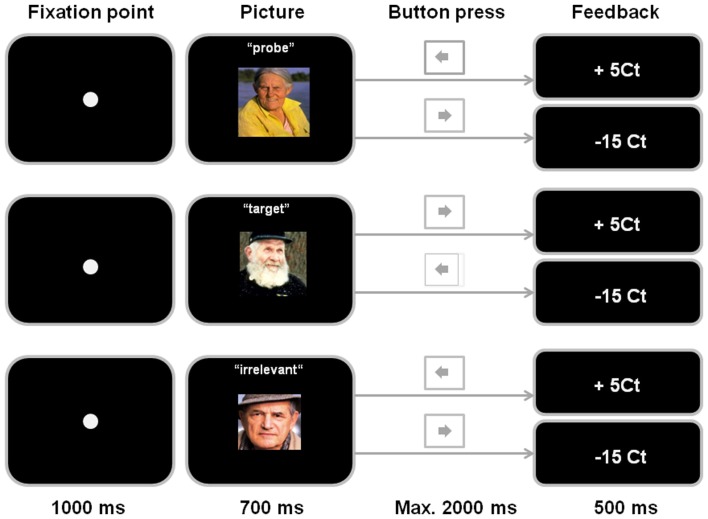
**Sequence of a probe item, a target item, and an irrelevant item**. The inter-trial-interval (ITI), which was 1000, 1500, or 2000 ms, is not presented in the figure.

Each trial consisted of a fixation point that was presented in the center of the TFT screen (20′′) for 1000 ms followed by a picture presented for 700 ms (picture size: 6 cm × 4 cm). Participants were instructed to indicate the picture type (probe, target, or irrelevant) by pressing the left-hand site cursor for a probe or an irrelevant picture and by pressing the right-hand site cursor for a target picture as soon as they were sure of the picture type. When a picture disappeared after 700 ms, participants could respond up to a maximum of 2000 ms. During this time interval the screen remained black. Correct responses to target pictures (i.e., pressing the right cursor button) and to irrelevant pictures (i.e., pressing the left cursor button) as well as successfully concealed knowledge to probe pictures (i.e., pressing the left cursor button) resulted in a win feedback (+5 Ct). Loss feedback (−15 Ct) occurred following each incorrect response (i.e., pressing the left cursor following a target picture and pressing the right cursor button to an irrelevant or probe picture). Moreover, following five out of 20 correct responses on probe items per block participants received a loss feedback (−15 Ct) even when they had pressed the left cursor button as required per instruction (cf. Fang et al., 2003). Participants always received a feedback that corresponded to the correctness of their responses in case of target and irrelevant pictures. Feedback to probe pictures corresponded to the correctness of their responses for 20 probe trials per block, whereas in five probe trials per block loss feedback (−15 Ct) occurred even when participants had correctly pressed the left cursor button (i.e., loss feedback was pre-defined). The sequence of a trial with a pre-defined loss feedback was as follows: participants saw a probe picture and responded to the left cursor button as they should for probes according to the instruction. Subsequently, they received a loss feedback of −15 Ct indicating that the computer program had detected that participants had concealed knowledge to the presented picture. This pre-defined loss feedback was realized in order to enhance the motivation of the participants to give their best in successfully concealing knowledge (Fang et al., 2003). The feedback was displayed for 500 ms on the screen (Figure 1). The inter-trial-interval (ITI) varied in a pseudo-random order between 1000, 1500, and 2000 ms. During ITI the screen remained black.

### Procedure

After arriving, participants gave written informed consent and were prepared for physiological recording. Participants were seated in a comfortable chair approximately 95 cm from the 20′′ computer TFT screen. The room was sound-attenuated and well-lit without dazzling the participants. Presentation V12.1 (Neurobehavioral Systems, Albany, NY, USA) was used to present the deception task. At the beginning of the task, participants learned the three pictures of the target category and the three pictures of the probe category for 5 min, whereas irrelevant pictures were not learned. Afterward participants performed 15 practice trials (including five probe, five irrelevant, and five target pictures). When responses to at least 12 pictures were correct, the main part of the deception task started. Otherwise the practice trials were repeated to make sure that participants were sufficiently familiar with the task. The deception task took on average 30 min (including learning and practice trials). The EEG was recorded during task performance. Each examination lasted about 1.5 h. At the end of the examination participants were thanked and paid depending on their performance (max. 15 EUR, about 20 USD).

### EEG recording

**Figure 2:**
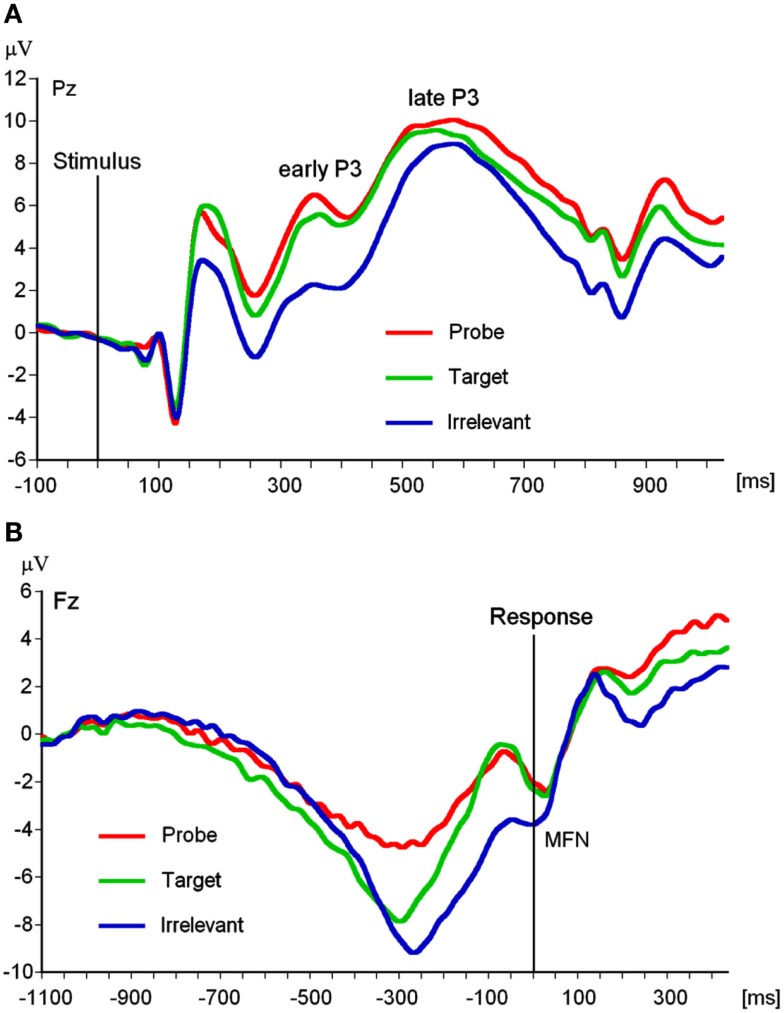
**(A)** Stimulus-locked grand averages at Pz separated for Picture type (*N* = 102). **(B)** Response-locked grand averages at Fz separated for Picture type (*N* = 91).

**Figure 3:**
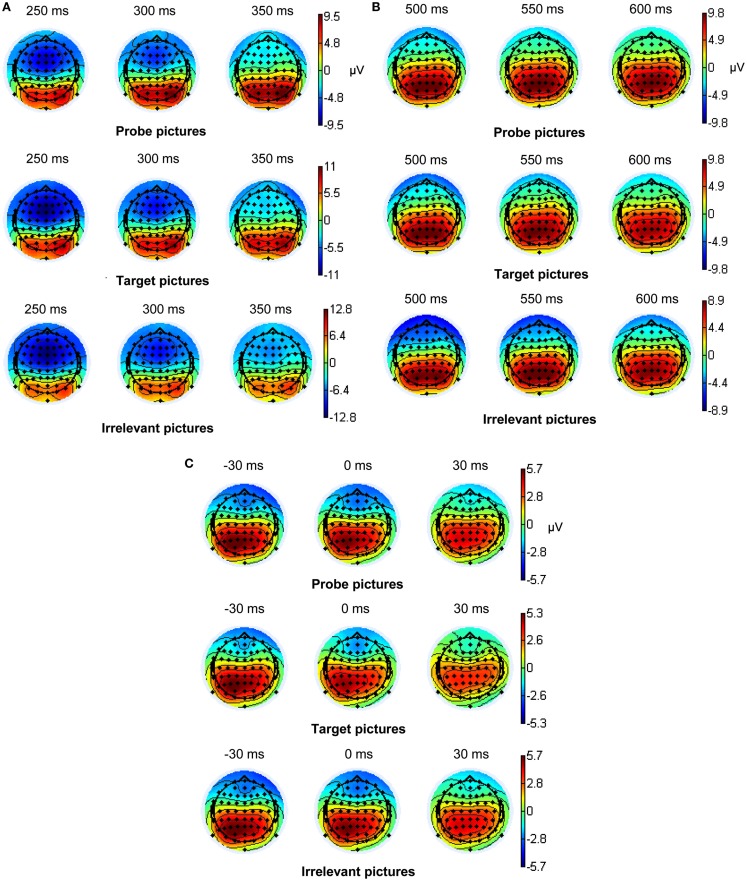
**(A)** Topographic maps of the early stimulus-locked P3 component (*N* = 102), **(B)** the late stimulus-locked P3 component, and **(C)** topographic maps of the response-locked MFN component (*N* = 91).

EEG recording, quantification, and analysis were conducted with reference to the guidelines for the study of human ERPs (Picton et al., 2000). The EEG was recorded using the ActiveTwo EEG system (BioSemi, Amsterdam, Netherlands) with 64 scalp active electrodes based on the extended 10/20 system (Jasper, 1958). The electrooculogram (EOG) was recorded from two horizontal electrodes placed beyond the epi canthi of both eyes and one vertical electrode located approximately 1 cm below the right eye. As per BioSemi’s design, the ground electrode during acquisition was formed by the Common Mode Sense active electrode and the Driven Right Leg passive electrode. All bioelectric signals were digitized on a laboratory computer using ActiView software (BioSemi). The impedances were below 30 kΩ during EEG recording. The EEG was sampled at 512 Hz. Off-line analysis was performed by using EEGLab v9.0.0.2 (Delorme and Makeig, 2004) based on MATLAB 7.10.0 (The MathWorks). All data were band-pass filtered (0.3–30 Hz) and were re-referenced to averaged mastoids (cf. Soskins et al., 2004 for filter settings in P300 studies). ICA (an automated infomax decomposition) was applied to correct for ocular artifacts. Further technical and muscle artifacts were rejected when the EEG signal exceeded ±85 μV. Artifact-free epochs with instruction-conform responses were separately segmented for the three picture types (probe, target, and irrelevant). Participants included into statistical analysis of the P3 components had at least 20 artifact-free epochs of each picture type (irrelevant: *M* = 40.65, SD = 9.99, target: *M* = 39.90, SD = 9.52, probe: *M* = 39.39, SD = 9.97) and for the MFN (irrelevant: *M* = 40.75, SD = 9.89, target: *M* = 37.73, SD = 7.37, probe: *M* = 39.16, SD = 9.55). Grand averages of the picture-related ERPs (0–1000 ms, with a 100 ms pre-stimulus baseline) indicate an early P3 amplitude between 280 and 350 ms post-stimulus and a late P3 amplitude between 440 and 610 ms post-stimulus (Figure 2A) both with a parietal topography (Figures 3A,B). The MFN (with 0 ms indicating the occurrence of the response) was identified between 0 and 40 ms post-response in a time window −1100 ms pre-response to 500 ms post-response with −1100 to −1000 ms serving as an ERP-neutral baseline (Figure 2B) and demonstrated a frontal topography (Figure 3C). The ERP components of interest were quantified as baseline-to-peak amplitudes (i.e., using the most positive peak for the P3 and the most negative peak for the MFN in the respective time interval). To correct for the influence of the positive ERP that occurred prior to the MFN we subtracted the positive peak of this preceding pre-response ERP from the MFN peak for each picture type and each electrode position included into statistical analysis.

### Statistical analysis

Using SPSS 18.0, repeated-measures ANOVAs were performed for behavioral and ERP data (i.e., stimulus-locked P3 amplitude and response-locked MFN amplitude). Picture type (probe, target, and irrelevant) was applied as a repeated-measures factor in ANOVA for behavioral and ERP data. In addition, Region (i.e., frontal sites collapsed across F3, Fz, F4; central sites collapsed across C3, Cz, C4; parietal sites collapsed across P3, Pz, P4) was applied as a repeated-measures factor in the ANOVA of ERP data. Repeated-measures ANOVAs were conducted with Gender, SI-perpetrator, and Trait-BIS as between-subjects factors. Participants were split into three personality subgroups based on percentiles. Individuals with personality scores below and equal to the 33rd percentile were classified as individuals with low personality scores (Trait-BIS ≤ 2.6: *N* = 38, SI-perpetrator ≤ 3.2: *N* = 41). Individuals with personality scores above the 33rd percentile and below or equal to the 66th percentile were classified as individuals with medium personality scores (Trait-BIS > 2.6 and ≤3.1: *N* = 30, SI-perpetrator >3.2 and ≤3.9: *N* = 31). Individuals with personality scores above the 66th percentile (Trait-BIS > 3.1: *N* = 34, SI-perpetrator > 3.9: *N* = 30) were classified as individuals with high scores.

Mean response times (RT) for the three picture categories were not normally distributed according to Kolmogorov–Smirnov test (*p* < 0.10). Therefore ln-transformed RT were applied to repeated-measures ANOVA (Wilkowski et al., 2010). The early and late P3 amplitudes (Kolmogorov–Smirnov test: *p* = 0.32–0.99) and the MFN-amplitudes (Kolmogorov–Smirnov test: *p* = 0.60–0.99) were normally distributed. For repeated-measures ANOVA, we report the uncorrected degrees of freedom along with Greenhouse–Geisser epsilons that indicate the violation of the sphericity assumption in the repeated-measures design. In addition to the significance level we report effect size eta square (η^2^). According to Cohen (1988) a small effect size is represented by an η^2^ of about 0.010, a medium effect size is given for an η^2^ of about 0.059, and a large effect size is represented by an η^2^ of about 0.138. In the Section “Discussion” we focus on those results that are of a large effect size.

In the present study the effect of the within-subjects factor Picture type on ERP-amplitudes was analyzed together with the between-subjects factors Trait-BIS and SI-perpetrator. In repeated-measures ANOVA the interactions of the within-subjects and between-subjects factors can only be calculated and traced back in further analyses when Trait-BIS- and SI-perpetrator-groups are formed in order to represent the between-subjects factors. Thus, the individual differences are reduced to those aspects that can be represented by the group variables. Even when we already formed three groups for each trait, this does not account for the complete variability of individual differences. In order to overcome this limitation of the repeated-measures ANOVA different methods have been proposed. For example, mixed-model ANOVA allows for a more complete representation of individual differences. However, only relative fit indices (Akaike Information Criterion, Bayesian Information Criterion) are available for mixed-model ANOVA (Liu et al., 2012), which might be regarded as a limitation of this approach. Another approach that allows for a complete representation of individual differences together with the interesting experimental effects, are ‘fixed-links’ models, which have been introduced by Schweizer (2006, 2008) on the basis of latent-growth models (Chan, 1998; Muthén and Muthén, 2010) in the context of SEM. The major characteristic that the fixed-links model shares with conventional growth models in the context of SEM is that the loadings of the latent variables are fixed according to specific hypotheses and that the variances of the latent variables are estimated. In contrast to conventional growth models based on SEM, fixed-links models allow for modeling of treatment effects that do not necessarily represent a temporal order (Schweizer, 2008). The first advantage of these models is that both the absolute fit of the models (e.g., χ^2^-test) and the relative fit of the models can be determined. The second advantage of the fixed-links models is that estimation methods are available that allow for parameter estimation even when there is a violation of the multivariate normal distribution in the data (Satorra and Bentler, 1994). The third advantage of fixed-links models is that, besides the modeling of experimental effects, they allow for an evaluation of the measurement models for the dependent variables, because the dependent variables can be represented by latent variables. Here, the dependent variables were the ERP-amplitudes that were represented by latent variables so that the measurement models for ERP-amplitudes were also evaluated. The fixed-links model has been successfully applied in different analyses of cognitive tasks (e.g., Miller et al., 2010). Because of the above-mentioned advantages, fixed-links models were calculated with Mplus 6.1 (Muthén and Muthén, 2010) in order to represent the complete variability of individual differences together with the treatment effects. In addition to the χ^2^-test, the Root Mean Square Error of Approximation (RMSEA), the Comparative Fit Index (CFI), and the standardized root mean square residual (SRMR) were reported in order to evaluate model fit.

## Results

### Behavioral data

A Picture type main effect was observed for the percentage of correct responses, *F*(2,176) = 35.79, *p* < 0.01, ε = 0.77, η^2^ = 0.29. Simple contrasts revealed that the percentage of correct responses was significantly lower to probe compared to irrelevant pictures, *F*(1,88) = 10.92, *p* < 0.01, η^2^ = 0.11, and to target compared to irrelevant pictures, *F*(1,88) = 48.52, *p* < 0.01, η^2^ = 0.36. The percentage of correct responses was significantly higher to probe than to target pictures, *F*(1,88) = 30.69, *p* < 0.01, η^2^ = 0.26 (Table 1).

**Table 1:** **Descriptive statistics of the number of correct responses and mean response times depending on picture type**.

Picture type	Percentage correct responses		Response times (ms)	
	*M*	SE	*M*	SE
Probe	97.65	0.31	730.35	24.85
Target	94.72	0.55	745.29	22.85
Irrelevant	99.50	0.13	690.23	23.01

**N* = 102. In purpose of simplicity response times are presented without ln-transformation in the table*.

Correct mean RT differed among Picture types, *F*(2,176) = 26.30, *p* < 0.01, ε = 0.93, η^2^ = 0.23. Simple contrasts revealed that RT were significantly longer for probe compared to irrelevant pictures, *F*(1,88) = 19.79, *p* < 0.01, η^2^ = 0.15, and for target compared to irrelevant pictures, *F*(1,88) = 58.69, *p* < 0.01, η^2^ = 0.40. RT to probe pictures were shorter than RTs to target pictures, *F*(1,88) = 4.65, *p* < 0.05, η^2^ = 0.05 (Table 1). There was no main effect of Trait-BIS or SI-perpetrator and no interaction of Picture type × SI-perpetrator or Picture type × Trait-BIS for number of correct responses and RT.

### P3 amplitude

**Figure 4:**
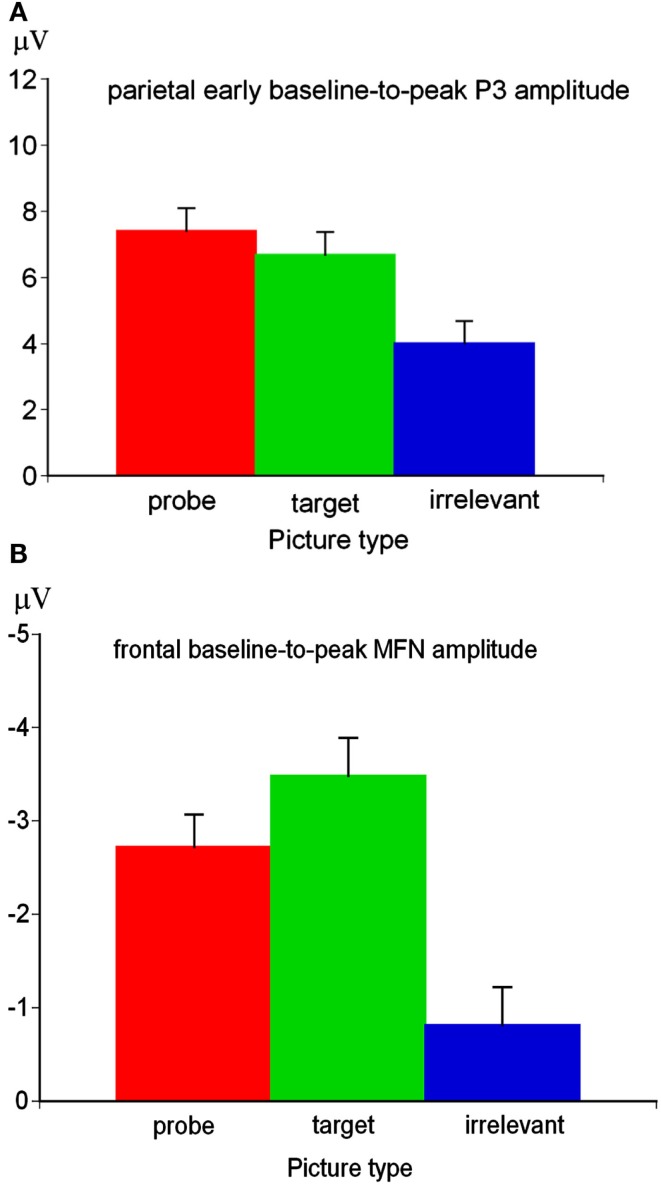
**Picture type main effect for the early parietal baseline-to-peak P3 amplitude (A) and picture type main effect of the frontal baseline-to-peak MFN amplitude (B)**.

The Region main effect of the early P3 amplitude was significant, *F*(2,176) = 127.57, *p* < 0.01, ε = 0.60, η^2^ = 0.59. Simple contrasts revealed a more positive P3 amplitude at parietal sites (*M* = 6.03 μV, SE = 0.65) compared to central electrode sites (*M* = −0.95 μV, SE = 0.82), *F*(1,88) = 142.34, *p* < 0.01, η^2^ = 0.62, and compared to frontal sites (*M* = −3.60 μV, SE = 0.90), *F*(1,88) = 135.96, *p* < 0.01, η^2^ = 0.61. Since the Region main effect indicated the typical parietal P3 topography, further analyses have been conducted for the early parietal P3. At parietal sites, the Picture type main effect was significant for the P3 amplitude, *F*(2,176) = 47.83, *p* < 0.01, ε = 0.90, η^2^ = 0.35. Simple contrasts indicated that the P3 amplitude was more positive for probe compared to irrelevant pictures, *F*(1,88) = 66.55, *p* < 0.01, η^2^ = 0.43, and for target compared to irrelevant pictures, *F*(1,88) = 54.10, *p* < 0.01, η^2^ = 0.38. The early P3 amplitude was also more positive for probe compared to target pictures, *F*(1,88) = 5.56, *p* < 0.05, η^2^ = 0.06 (Figure 4A).

**Figure 5:**
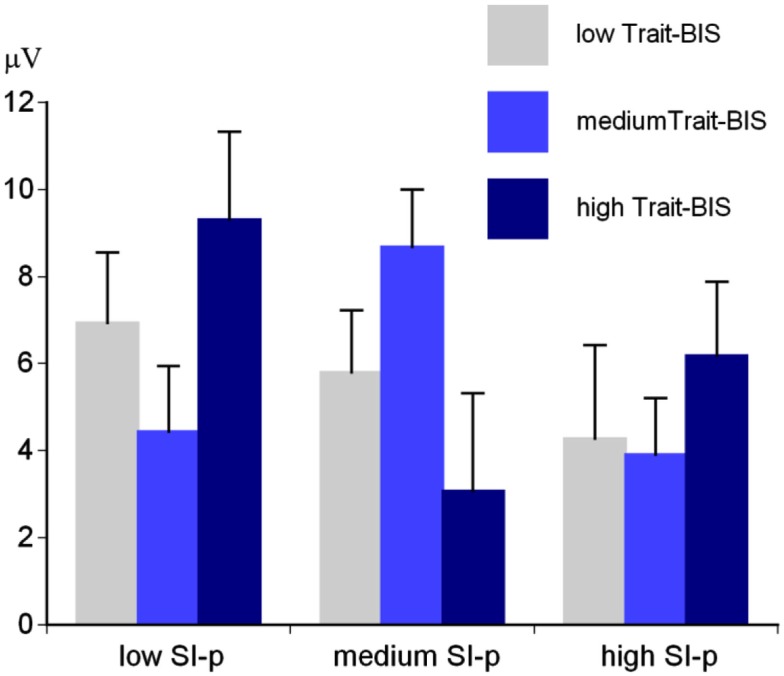
**Early baseline-to-peak P3 amplitude for SI-perpetrator (SI-p) scores × Trait-BIS scores (low Trait-BIS/low SI-p: *N* = 18; low Trait-BIS/medium SI-p: *N* = 13; low Trait-BIS/high SI-p: *N* = 7; medium Trait-BIS/low SI-p: *N* = 10; medium Trait-BIS/medium SI-p: *N* = 10; medium Trait-BIS/high SI-p: *N* = 10; high Trait-BIS/low SI-p: *N* = 13; high Trait-BIS/medium SI-p: *N* = 8; high Trait-BIS/high SI-p: *N* = 13)**.

Regarding personality, there was a significant SI-perpetrator ×  Trait-BIS interaction for the early parietal P3 amplitude, *F*(4,88) = 2.71, *p* < 0.05, η^2^ = 0.11. This interaction could be traced back to a significant SI-perpetrator main effect for individuals with medium Trait-BIS scores, *F*(2,24) = 3.90, *p* < 0.05, η^2^ = 0.25 (Figure 5). Individuals with medium SI-perpetrator and medium Trait-BIS scores showed the more positive early parietal P3 amplitude (*M* = 8.68 μV, SE = 1.31) compared to individuals with low SI-perpetrator and medium Trait-BIS scores (*M* = 4.44 μV, SE = 1.50) and individuals with high SI-perpetrator and medium Trait-BIS scores (*M* = 3.90 μV, SE = 1.31).

For the late P3 amplitude, a significant Region main effect was observed, *F*(2,176) = 122.62, *p* < 0.01, ε = 0.60, η^2^ = 0.58. As for the early P3 amplitude, simple contrasts indicated a more positive late parietal P3 amplitude (*M* = 9.80 μV, SE = 0.67) compared to the central P3 amplitude (*M* = 6.15 μV, SE = 0.71), *F*(1,88) = 71.22, *p* < 0.01, η^2^ = 0.45. Again, because of the parietal P3 topography, the Picture type main effect for the late P3 amplitude was analyzed at parietal sites, *F*(2,176) = 4.31, *p* < 0.05, ε = 0.93, η^2^ = 0.05. Simple contrasts suggested a more positive late P3 amplitude for probe pictures (*M* = 10.24 μV, SD = 0.69) compared to irrelevant pictures (*M* = 9.17 μV, SD = 0.69), *F*(1,88) = 7.17, *p* < 0.01, η^2^ = 0.08, and for target pictures (*M* = 10.00 μV, SD = 0.72) compared to irrelevant pictures, *F*(1,88) = 3.96, *p* = 0.05, η^2^ = 0.04. In contrast to the early parietal P3 amplitude, the late P3 amplitude of probe compared to target pictures did not substantially differ, *F*(1,88) < 1, ns. Also in contrast to the early P3, the SI-perpetrator × Trait-BIS interaction was only marginally significant for the late parietal P3 amplitude, *F*(4,88) = 2.32, *p* = 0.06, η^2^ = 0.10. As for the early parietal P3, individuals with medium SI-perpetrator and with medium Trait-BIS scores showed the most positive late parietal P3 amplitude, *F*(2,24) = 4.24, *p* < 0.05, η^2^ = 0.26. The Pearson correlations between the early P3 amplitude and the late P3 amplitude were 0.63 at Pz, 0.69 at P3, and 0.64 at P4 (*N* = 102, all *p*s < 0.01, two-tailed). Thus, the parietal early and late P3 amplitudes were significantly correlated. It should also be noted that both the early P3 and the late P3 have a parietal topography so that the early P3 amplitude should probably not be regarded as a P3a or novelty P3, which is known to have a frontal topography (Kok, 2001).

### MFN amplitude

The Region main effect of the MFN amplitude was significant, *F*(2,154) = 12.94, *p* < 0.01, ε = 0.67, η^2^ = 0.14. Simple contrasts indicated a more negative MFN amplitude at frontal sites (*M* = −2.52 μV, SE = 0.35) compared to central sites (*M* = −1.04 μV, SE = 0.34), *F*(1,77) = 44.45, *p* < 0.01, η^2^ = 0.37, and at parietal sites (*M* = −2.00 μV, SE = 0.31) compared to central sites, *F*(1,77) = 14.32, *p* < 0.01, η^2^ = 0.16, but not at frontal compared to parietal sites, *F*(1,77) = 1.83, *p* = 0.18. In order to investigate variations of cognitive control, further analyses focused on the frontal MFN. The Picture type main effect of the frontal MFN amplitude was significant, *F*(2,148) = 27.14, *p* < 0.01, ε = 0.94, η^2^ = 0.27. Simple contrasts indicated that the target-MFN was more negative than the probe-MFN, *F*(1,74) = 6.02, *p* < 0.05, η^2^ = 0.08. The MFN amplitude was more negative for target pictures compared to irrelevant pictures, *F*(1,74) = 41.83, *p* < 0.01, η^2^ = 0.36, and more importantly for probe compared to irrelevant pictures, *F*(1,74) = 28.37, *p* < 0.01, η^2^ = 0.28 (Figure 4B). The Picture type × SI-perpetrator interaction, *F*(4,148) < 1, ns, the Picture type × Trait-BIS interaction, *F*(4,148) < 1, ns, and the Picture type × SI-perpetrator × Trait-BIS interaction, *F*(8,148) = 1.39, ns, were not significant.

### Fixed-links modeling

**Figure 6:**
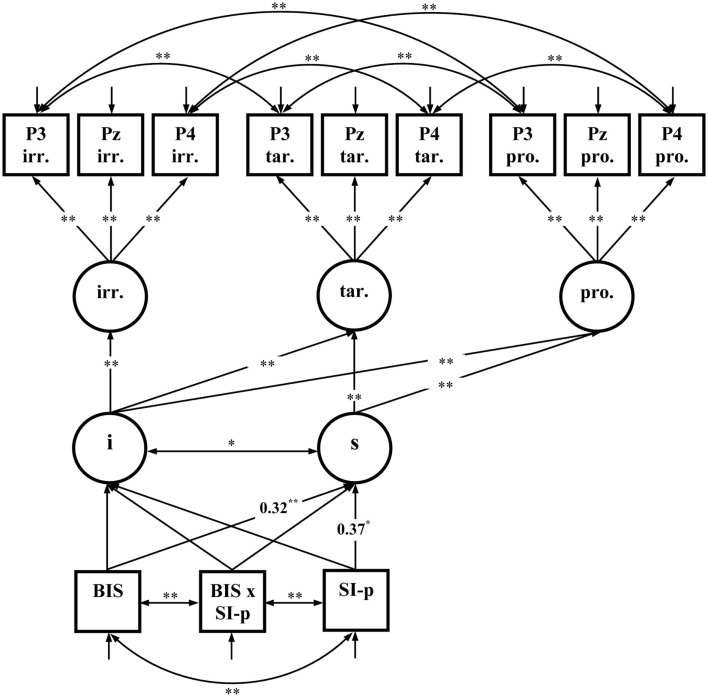
Fixed-links model for early P3 amplitudes across picture types (*N* = 85); i, intercept; s, slope; irr., irrelevant pictures; tar., target pictures; pro., probe pictures; significant coefficients are marked with “*” (*p* ≤ 0.05, two-tailed) and “**” (*p* ≤ 0.01, two-tailed). For convenience, numerical values of the completely standardized solution are only given for significant coefficients related to Trait-BIS (BIS), SI-perpetrator (SI-p), and the Trait-BIS × SI-perpetrator interaction (BIS × SI-p).

The first fixed-links model comprised latent variables representing the early P3 amplitudes for each Picture Type (irrelevant, target, and probe) at three relevant electrode sites (P3, Pz, and P4; see Figure 6). Residuals were allowed to correlate for electrode sites P3 and P4 indicating common variance of these electrode positions. Measurement invariance was specified by holding the means and factor loadings of the factor indicators equal across picture types. The intercept and the linear slope for the increase of the latent variables representing P3 amplitudes from irrelevant, to target, and probe pictures was calculated. The slope represents the effects of Picture type on P3 amplitudes and was predicted by Trait-BIS, SI-perpetrator, and the Trait-BIS × SI-perpetrator interaction. Since the multivariate normal distribution was not given for the variables included into the model $\chi_{\left(2\right)}^{2}$ = 181.42; *p* < 0.01 the robust maximum-likelihood estimation was performed and the Satorra–Bentler scaled $\chi_{\text{SB}}^{2}$ statistic (Satorra and Bentler, 1994) was reported. The model fits quite well to the data $\chi_{\text{SB}\left(54\right)}^{2}$ = 66.30; *p* = 0.12; RMSEA = 0.052; CFI = 0.99; SRMR = 0.060. Trait-BIS and SI-perpetrator were significant positive predictors of the slope of the early P3 amplitudes (see Figure 6). The positive predictions of the slope indicate that the increase of the early P3 amplitude from irrelevants over targets to probes is more substantial for individuals with higher Trait-BIS scores as well as for individuals with higher SI-perpetrator scores.

The second fixed-links model comprised latent variables representing the late P3 amplitudes for each Picture type (irrelevant, target, and probe) at three electrode sites (P3, Pz, and P4). The model was specified like the previous model so that an additional figure would have been redundant. Since the variables included deviate from the multivariate normal distribution $\chi_{\left(2\right)}^{2}$ = 275.75; *p* < 0.01 robust maximum-likelihood estimation was performed and the Satorra–Bentler scaled $\chi_{\text{SB}}^{2}$ statistic was reported. The model fits very well to the data $\chi_{\text{SB}\left(53\right)}^{2}$ = 61.99; *p* = 0.98; RMSEA = 0.045; CFI = 0.99; SRMR = 0.038. However, there were no effects for personality on the late P3 amplitudes.

**Figure 7:**
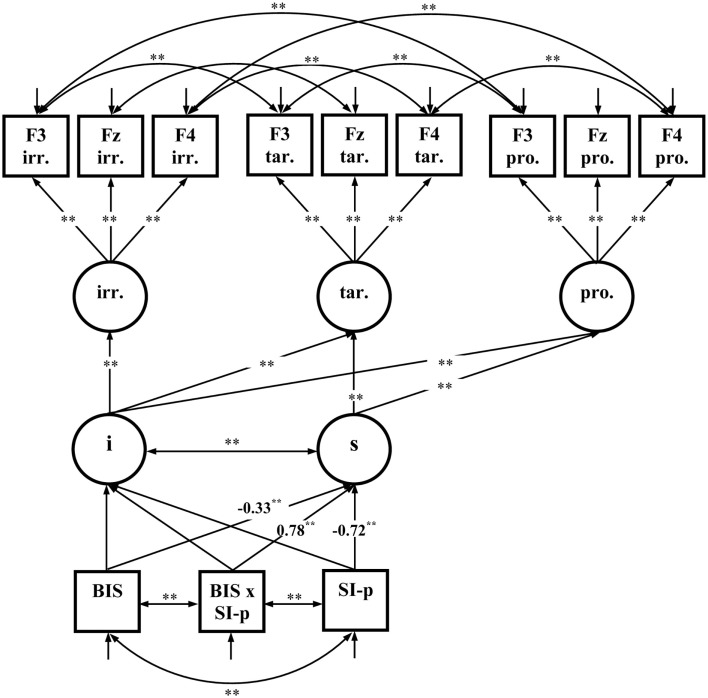
Fixed-links model for MFN-amplitudes across picture types (*N* = 85); i, intercept; s, slope; irr., irrelevant pictures; tar., target pictures; pro., probe pictures; significant coefficients are marked with “*” (*p* ≤ 0.05, two-tailed) and “**” (*p* ≤ 0.01, two-tailed). For convenience, numerical values of the completely standardized solution are only given for significant coefficients related to Trait-BIS (BIS), SI-perpetrator (SI-p), and the Trait-BIS × SI-perpetrator interaction (BIS × SI-p).

The third fixed-links model comprised latent variables representing the MFN-amplitudes for each Picture Type (irrelevant, target, and probe) at three relevant electrode sites (F3, Fz, F4). Residuals were allowed to correlate for electrode sites indicating common variance due to electrode positions. Measurement invariance was specified by holding the means and factor loadings of the factor indicators equal across picture types. The intercept and the linear slope for the decrease of the latent variables representing MFN-amplitudes from irrelevant, to target, and probe pictures was calculated. The slope represents the effects of Picture type on MFN-amplitudes. Again, the multivariate normal distribution was not given for the variables included into the model $\chi_{\left(2\right)}^{2}$ = 218.02; *p* < 0.01 so that robust maximum-likelihood estimation was performed and the Satorra–Bentler scaled $\chi_{\text{SB}}^{2}$ statistic was reported. The model fits well to the data $\chi_{\text{SB}\left(52\right)}^{2}$ = 61.39; *p* = 0.17; RMSEA = 0.046; CFI = 0.99; SRMR = 0.073. There were significant negative path coefficients from Trait-BIS and SI-perpetrator to the MFN-slope indicating that individuals with higher Trait-BIS and SI-perpetrator scores have a more pronounced decrease of MFN-amplitudes from irrelevant, to target, and probe pictures (see Figure 7). Moreover, there is a significant positive path coefficient from the Trait-BIS × SI-perpetrator interaction to the MFN-slope indicating that individuals with both higher Trait-BIS and higher SI-perpetrator scores had a less pronounced decrease of MFN-amplitudes.

## Discussion

The present study investigated individual differences of Trait-BIS and SI-perpetrator with regard to stimulus salience, attentional control, and cognitive control in a visual deception task. Stimulus salience and attentional control were investigated by means of the stimulus-locked P3 amplitude and cognitive control was investigated by means of the response-locked MFN. The main ERP findings with strong effect sizes are: (a) According to ANOVA and fixed-links modeling, the parietal P3 amplitudes were more positive to probe and target pictures compared to irrelevant pictures. (b) Fixed-links modeling results indicated that higher Trait-BIS as well as higher SI-perpetrator scores were related to a more pronounced early P3 increase from irrelevant to target and to probe pictures. (c) The response-locked frontal MFN amplitude was more negative for probe and target compared to irrelevant pictures. (d) Fixed-links modeling demonstrated that higher Trait-BIS scores as well as higher SI-perpetrator scores predicted a more pronounced MFN decrease from irrelevant to probe pictures. However, the Trait-BIS × SI-perpetrator interaction in the fixed-links model suggested a smaller MFN decrease from irrelevant to probe pictures for individuals with both higher Trait-BIS and higher SI-perpetrator scores. We discuss the implications of these main findings subsequently.

### Variations of stimulus salience and attentional control

Our P3 results in a reinforcement-related deception task support findings of prior deception studies showing more pronounced parietal P3 amplitudes to probe compared to irrelevant pictures (e.g., Mertens and Allen, 2008; Ambach et al., 2010). From the perspective of the salience hypothesis (Kok, 2001), the present findings suggest that irrelevant pictures are less salient (resulting in smaller P3 amplitudes) than probe and target pictures. In this line and in accordance with prior studies longer RT were observed for probe stimuli compared to irrelevant stimuli (Walczyk et al., 2003; Dong et al., 2010b). This suggests that participants are more sensitive and subsequently more cautious in responding to probe pictures compared to irrelevant pictures supporting the salience hypothesis. Moreover, based on the attentional control approach it cannot be excluded that RT to probes were slower because more attentional and/or processing resources were needed to inhibit the primary task of responding truthfully (e.g., Johnson et al., 2003). As a new finding we could demonstrate by means of fixed-links modeling that deceiving knowledge is more salient for higher vs. lower Trait-BIS individuals and also for higher vs. lower SI-perpetrator individuals because the early P3 amplitude increased from irrelevant to probe pictures for both personality dimensions (Figure 6). Our results suggest that deceiving knowledge is more salient (resulting in a larger probe-P3) for those individuals who show an increased sensitivity to aversiveness (higher Trait-BIS) and those individuals who are more sensitive toward situations in that they treat others unfairly (higher SI-perpetrator).

### Variations of cognitive control

In our study the variations of the MFN illustrate that probe compared to target and irrelevant pictures require more cognitive control. Our MFN findings correspond to prior studies in that the probe-MFN was more negative than the irrelevant-MFN (Dong et al., 2010a). Because this finding of a more negative probe-MFN compared to irrelevant-MFN parallels to MFN findings in non-deception studies illustrating a more negative MFN to erroneous compared to correct responses (Luu et al., 2000; Potts et al., 2006), one might conclude that erroneous as well as deceptive responses are more aversive and this might also contribute to an increase in cognitive control. Moreover, the decrease of the MFN from irrelevant to target and to probe pictures was more pronounced in higher vs. lower Trait-BIS individuals and in higher vs. lower SI-perpetrator individuals. This finding illustrates that individuals who have either higher Trait-BIS scores or higher SI-perpetrator scores invest more cognitive control in their responses to probe items. Since both trait-dimensions were positively correlated we presume that they share variance in aversiveness sensitivity. Therefore, we conclude with regard to the revised reinforcement sensitivity theory (Corr, 2008) that deceiving knowledge is not only more salient (see P3 findings) but also evokes a more pronounced investment of cognitive control (see MFN findings). The more pronounced MFN of higher vs. lower Trait-BIS individuals corresponds to ERN findings in non-deception studies (e.g., Boksem et al., 2006) with higher Trait-BIS individuals showing more negative ERN amplitudes. This indicates that erroneous and deceptive responses share cognitive processes that are activated in the Anterior Cingulate Cortex (cf. Johnson et al., 2004, 2008). Moreover, our MFN findings suggest that a combination of higher SI-perpetrator and higher Trait-BIS scores reduces the amount of cognitive control invested to probes. This could be due to the fact that resources for response-related control might be still occupied by moral justification in these individuals. Overall, the results indicate that salience processes (P3) as well as cognitive control processes (MFN) co-occur in a deception task.

### Limitations and future directions

Dipole modeling in a deception setting (cf. Johnson et al., 2008) might be promising to further investigate the functioning of the fronto-parietal network during executive control that has been described in imaging studies (e.g., Christ et al., 2009). Moreover, our data suggest that both stimulus- and response-locked ERPs are promising in order to differentiate deceptive vs. truthful knowledge. Therefore, future research could clarify whether the combination of different ERPs contributes to more correct classifications of truthful vs. deceptive knowledge in guilty compared to innocent persons. Recent findings demonstrate that enhanced emotional arousal assessed by heart rate changes from baseline to experimental task was observed after committing a mock crime in the context of a CIT. Moreover, enhanced emotional arousal reduced memory of peripheral information in the CIT (Peth et al., 2012). Since individual differences like trait-anxiety or trait-BIS have been associated with an increased arousal (e.g., Gray and McNaughton, 2000) it might be interesting to investigate individual differences of trait-anxiety or trait-BIS with our deception task under different arousal conditions. Moreover, individual differences of Trait-BIS and SI-perpetrator predicted variations of the P3 and the MFN so that both trait-dimensions appear to be promising moderators for the classification of guilty vs. innocent individuals in CIT. By using 3 different probes, 20 different irrelevants, and 3 different target pictures we realized the traditional stimulus ratio applied in prior deception studies (e.g., Meijer et al., 2007). However, since each picture type occurred with the same total frequency, it remains for further clarification whether this has an effect on the P3 and MFN findings. It remains also for replication whether aspects of stimulus salience and attentional control can be related to different P3 components.

It should be noted that the effects of personality were found for the early P3 amplitude but not for the later P3 amplitude. At this point we can only speculate on the reasons for this result. One possibility could be that the more early P3 amplitude reflects a more spontaneous and therefore a more affective aspect of stimulus processing, whereas the later P3 amplitude is related to subsequent, more cognitive processes. Finally, despite applying a 0.3 Hz high-pass filter the late P3 component does not entirely return to the baseline level 1 s after stimulus-onset (for similar observations see Fang et al., 2003; Ambach et al., 2010; Gamer and Berti, 2010). At this point of research we cannot exclude whether variable ITIs or variations in sampling rate might account for this phenomenon. According to Soskins et al. (2004) we can also not completely exclude that the negative waveform following the late P3 could be a distorted post-peak recovery of P3.

Based on the present findings we draw the following conclusions: First, parietal P3 and frontal MFN are ERPs that are related to an intensification of stimulus salience and cognitive control in a deception task. P3 and MFN became more pronounced from irrelevant to target and to probe stimuli. Second, Trait-BIS and SI-perpetrator modulate the intensity of stimulus salience (early P3) and cognitive control (MFN) in a deception task, whereas behavioral parameters were not sensitive to personality differences. Third, our data encourage the simultaneous investigation of stimulus-locked and response-locked ERPs to further elucidate patterns of neuro-cognitive processes during deception.

## Conflict of Interest Statement

The authors declare that the research was conducted in the absence of any commercial or financial relationships that could be construed as a potential conflict of interest.

## References

[B1] Allen J. J. Iacono W. G. Danielson K. D. (1992). The identification of concealed memories using the event-related potential and implicit behavioral measures: a methodology for prediction in the face of individual differences. Psychophysiology 29, 504–522 10.1111/j.1469-8986.1992.tb02024.x 1410180

[B2] Ambach W. Stark R. Peper M. Vaitl D. (2010). An interfering go/no-go task does not affect accuracy in a concealed information test. Int. J. Psychophysiol. 75, 258–267 10.1016/j.ijpsycho.2009.12.007 18180065

[B3] Amodio D. M. Master S. L. Yee C. M. Taylor S. E. (2008). Neurocognitive components of the behavioral inhibition and activation systems: implications for theories of self-regulation. Psychophysiology 45, 11–19 17910730 10.1111/j.1469-8986.2007.00609.x

[B4] Beauducel A. Brocke B. Leue A. (2006). Energetical bases of extraversion: effort, arousal, and vigilance performance. Int. J. Psychophysiol. 62, 212–223 10.1016/j.ijpsycho.2005.12.001 16426692

[B5] Boksem M. A. S. De Cremer D. (2010). Fairness concerns predict medial frontal negativity amplitude in ultimatum bargaining. Soc. Neurosci. 5, 118–128 10.1080/17470910903202666 19851940

[B6] Boksem M. A. S. Tops M. Wester A. E. Meijam T. F. Lorist M. M. (2006). Error-related ERP components and individual differences in punishment and reward sensitivity. Brain Res. 1101, 92–101 10.1016/j.brainres.2006.05.004 16784728

[B7] Bradley M. M. Lang P. J. (2007). “The international affective pictures system (IAPS) in the study of emotion and attention,” in Handbook of Emotion Elicitation and Assessment, eds Coan J. A. Allen J. B. (New York: Oxford University Press), 29–46

[B8] Carrión R. E. Keenan J. P. Sebanz N. (2010). A truth that’s told with bad intent: an ERP study of deception. Cognition 114, 105–110 10.1016/j.cognition.2009.05.014 19836013

[B9] Carver C. S. White T. L. (1994). Behavioral inhibition, behavioral activation, and affective responses to impending reward and punishment: the BIS/BAS scales. J. Pers. Soc. Psychol. 67, 319–333 10.1037/0022-3514.67.2.319

[B10] Chan D. (1998). The conceptualization and analysis of change over time: an integrative approach incorporating longitudinal mean and covariance structures analysis (LMACS) and multiple indicator latent growth modeling (MLGM). Organ Res. Methods 1, 421–483 10.1177/109442819814004

[B11] Christ S. E. Van Essen D. C. Watson J. M. Brubaker L. E. McDermott K. B. (2009). The contributions of prefrontal cortex and executive control to deception: evidence from activation likelihood estimate metaanalyses. Cereb. Cortex 19, 1557–1566 10.1093/cercor/bhn189 18980948 PMC2693617

[B12] Cohen J. (1988). Statistical Power Analysis for the Behavioral Sciences. Hillsdale, NJ: Lawrence Erlbaum Associates

[B13] Corr P. J. (2008). The Reinforcement Sensitivity Theory. Cambridge: Cambridge University Press

[B14] Cronbach L. J. (1975). Beyond the two disciplines of scientific psychology. Am. Psychol. 30, 116–127 10.1037/h0076829

[B15] Delorme A. Makeig S. (2004). EEGLAB: an open source toolbox for analysis of single-trial EEG dynamics including independent component analysis (sccn.ucsd.edu/eeglab/). J. Neurosci. Methods 134, 9–21 10.1016/j.jneumeth.2003.10.009 15102499

[B16] DePaulo B. M. Lindsay J. J. Malone B. E. Muhlenbruck L. Charlton K. Cooper H. (2003). Cues to deception. Psychol. Bull. 129, 74–118 10.1037/0033-2909.129.1.74 12555795

[B17] Dong G. Hu Y. Lu Q. Wu H. (2010a). The presentation order of cue and target matters in deception study. Behav. Brain Funct. 6, 1–9 10.1186/1744-9081-6-63 PMC2972230 20964866

[B18] Dong G. Wu H. Lu Q. (2010b). Attempting to hide our real thoughts: electrophysiological evidence from truthful and deceptive responses during evaluation. Neurosci. Lett. 479, 1–5 10.1016/j.neulet.2010.05.014 20470861

[B19] Dong G. Hu Y. Wu H. (2011). The presentation order of cue and target matters in deception study. Behav. Brain Funct. 7, 36 10.1186/1744-9081-6-63 PMC2972230 20964866

[B20] Fang F. Liu Y. Shen Z. (2003). Lie detection with contingent negative variation. Int. J. Psychophysiol. 50, 247–255 10.1016/S0167-8760(03)00170-3 14585493

[B21] Farwell L. A. Donchin E. (1991). The truth will out: interrogative polygraphy (“Lie detection”) with event-related brain potentials. Psychophysiology 28, 531–547 10.1111/j.1469-8986.1991.tb01990.x 1758929

[B22] Fink A. Neubauer A. C. (2004). Extraversion and cortical activation: effects of task complexity. Pers. Individ. Dif. 36, 333–347 10.1016/S0191-8869(03)00100-4

[B23] Gamer M. Berti S. (2010). Task relevance and recognition of concealed information have different influences on electrodermal activity and event-related brain potentials. Psychophysiology 47, 355–364 10.1111/j.1469-8986.2009.00933.x 20003148

[B24] Gombos V. A. (2006). The cognition of deception: the role of executive processes in producing lies. Genet. Soc. Gen. Psychol. Monogr. 132, 197–214 10.3200/MONO.132.3.197-214 17969998

[B25] Gray J. A. McNaughton N. (2000). The Neuropsychology of Anxiety. Oxford: University Press

[B26] Jasper H. H. (1958). The ten-twenty electrode system of the International Federation. Electroencephalogr. Clin. Neurophysiol. 10, 371–375 10590970

[B27] Johnson R. Jr. Barnhardt J. Zhu J. (2003). The deceptive response: effects of response conflict and strategic monitoring on the late positive component and episodic memory-related brain activity. Biol. Psychol. 64, 217–253 10.1016/j.biopsycho.2003.07.006 14630405

[B28] Johnson R. Jr. Barnhardt J. Zhu J. (2004). The contribution of executive processes to deceptive responding. Neuropsychologia 42, 878–901 10.1016/j.neuropsychologia.2003.12.005 14998703

[B29] Johnson R. Jr. Henkell H. Simon E. Zhu J. (2008). The self in conflict: the role of executive processes during truthful and deceptive responses about attitudes. Neuroimage 39, 469–482 10.1016/j.neuroimage.2007.08.032 17919934

[B30] Kok A. (2001). On the utility of P3 amplitude as a measure of processing capacity. Psychophysiology 38, 557–577 10.1017/S0048577201990559 11352145

[B31] Lange S. Leue A. Beauducel A. (2012). Behavioral approach and reward processing: results on feedback-related negativity and P3 component. Biol. Psychol. 89, 416–425 10.1016/j.biopsycho.2011.12.004 22178442

[B32] Leue A. Beauducel A. (2008). A meta-analysis of reinforcement sensitivity theory: on performance parameters in reinforcement tasks. Pers. Soc. Psychol. Rev. 12, 353–369 10.1177/1088868308316891 18544711

[B33] Leue A. Chavanon M.-L. Wacker J. Stemmler G. (2009). On the differentiation of N2 components in an appetitive choice task: evidence for the revised Reinforcement Sensitivity Theory. Psychophysiology 46, 1244–1257 10.1111/j.1469-8986.2009.00872.x 19674394

[B34] Leue A. Lange S. Beauducel A. (2012a). Modulation of the conflict monitoring intensity: the role of aversive reinforcement, cognitive demand, and trait-BIS. Cogn. Affect. Behav. Neurosci. 12, 287–307 10.3758/s13415-012-0086-x 22351495

[B35] Leue A. Lange S. Beauducel A. (2012b). Reinforcement sensitivity and conflict processing: a study of principal components in the N2 time domain. J. Individ. Dif. 33, 160–168

[B36] Liu S. Rovine M. J. Molenaar P. C. M. (2012). Selecting a linear mixed model for longitudinal data: repeated measures analysis of variance, covariance pattern model, and growth curve approaches. Psychol. Methods 17, 15–30 10.1037/a0026971 22251268

[B37] Luu P. Flaisch T. Tucker D. M. (2000). Medial frontal cortex in action monitoring. J. Neurosci. 20, 464–469 10627622 10.1523/JNEUROSCI.20-01-00464.2000 PMC6774138

[B38] Mecklinger A. Kramer A. F. Strayer D. L. (1992). Event related potentials and EEG components in a semantic memory search task. Psychophysiology 29, 104–119 10.1111/j.1469-8986.1992.tb02021.x 1609022

[B39] Meijer E. H. Smulders F. T. Y. Merckelbach H. L. G. J. Wolf A. G. (2007). The P300 is sensitive to concealed face recognition. Int. J. Psychophysiol. 66, 231–237 10.1016/j.ijpsycho.2007.08.001 17825933

[B40] Meijer E. H. Smulders F. T. Y. Wolf A. G. (2009). The contribution of mere recognition to the P300 effect in a concealed information test. Appl. Psychophysiol. Biofeedback 34, 221–226 10.1007/s10484-009-9099-9 19585234 PMC2727362

[B41] Meixner J. B. Rosenfeld J. P. (2011). A mock terrorism application of the P300-based concealed information test. Psychophysiology 48, 149–154 10.1111/j.1469-8986.2010.01050.x 20579312

[B42] Mertens R. Allen J. B. (2008). The role of psychophysiology in forensic assessments: deception detection, ERPs, and virtual reality mock crime scenarios. Psychophysiology 45, 286–298 10.1111/j.1469-8986.2007.00615.x 17995914

[B43] Miller R. Rammsayer T. H. Schweizer K. Troche S. J. (2010). Decay of iconic memory traces is related to psychometric intelligence: a fixed-links modeling approach. Learn. Individ. Differ. 20, 699–704 10.1016/j.lindif.2010.08.010

[B44] Mulder G. (1986). “The concept and measurement of mental effort,” in Energetics and Human Information Processing, eds Hockey R. J. Gaillard A. W. K. Coles M. G. H. (Dordrecht: Martinus Nijhoff), 175–198

[B45] Muthén B. O. (2004). “Latent variable analysis: growth mixture modeling and related techniques for longitudinal data,” in Handbook of Quantitative Methodology for the Social Sciences, ed. Kaplan D. (Newbury Park, CA: Sage Publications), 345–368

[B46] Muthén L. K. Muthén B. O. (2010). MPlus User’s Guide. Los Angeles, CA: Muthén & Muthén

[B47] Oldfield R. C. (1971). The assessment and analysis of handedness: the Edinburgh inventory. Neuropsychologia 9, 97–113 10.1016/0028-3932(71)90067-4 5146491

[B48] Peth J. Vossel G. Gamer M. (2012). Emotional arousal modulates the encoding of crime-related details and corresponding physiological responses in the concealed information test. Psychophysiology 49, 381–390 10.1111/j.1469-8986.2011.01313.x 22188567

[B49] Picton T. W. Bentin S. Berg P. Donchin E. Hillyard S. A. Johnson R. (2000). Guidelines for using human event-related potentials to study cognition: recording standards and publication criteria. Psychophysiology 37, 127–152 10.1111/1469-8986.3720127 10731765

[B50] Polich J. (2007). Updating P300: an integrative theory of P3a and P3b. Clin. Neurophysiol. 118, 2128–2148 10.1016/j.clinph.2007.04.019 17573239 PMC2715154

[B51] Potts G. Martin L. Burton P. Montague P. (2006). When things are better or worse than expected: medial frontal cortex and the allocation of processing resources. J. Cogn. Neurosci. 18, 1–8 10.1162/jocn.2006.18.7.1112 16839285

[B52] Raudenbush S. W. Bryk A. S. (2002). Hierarchical Linear Models: Applications and Data Analysis Methods. Thousand Oaks: CA: Sage

[B53] Rosenfeld J. P. Cantwell G. Nasman V. T. Wojdac V. Ivanov S. Mazzeri L. (1988). A modified event-related potential-based guilty knowledge test. Int. J. Neurosci. 42, 157–161 10.3109/00207458808985770 3209369

[B54] Rosenfeld J. P. Angell A. Johnson M. M. Qian J. H. (1991). An ERP-based, control-question lie detector analog: algorithms for discriminating effects within individuals’ average waveforms. Psychophysiology 28, 319–335 10.1111/j.1469-8986.1991.tb02202.x 1946897

[B55] Satorra A. Bentler P. M. (1994). “Corrections to test statistics and standard errors in covariance structure analysis,” in Latent Variables Analysis: Applications for Developmental Research, eds Von Eye A. Clogg C. C. (Thousand Oaks, CA: Sage), 399–419

[B56] Schmitt M. Gollwitzer M. Maes J. Arbach D. (2005). Justice sensitivity: assessment and location in the personality space. Eur. J. Psychol. Assess. 21, 202–211 10.1027/1015-5759.21.3.202

[B57] Schweizer K. (2006). The fixed-links model in combination with the polynomial function as a tool for investigating choice reaction time data. Struct. Equ. Modeling 13, 403–419 10.1207/s15328007sem1303_4

[B58] Schweizer K. (2008). Investigating experimental effects within the framework of structural equation modeling: an example with effects on both error scores and reaction times. Struct. Equ. Modeling 15, 327–345 10.1080/10705510801922621

[B59] Soskins M. Rosenfeld J. P. Niendam T. (2004). Peak-to-peak measurement of P300 recorded at 0.3 Hz high pass filter settings in intraindividual diagnosis: complex vs. simple paradigms. Int. J. Psychophysiol. 40, 173–180 10.1016/S0167-8760(00)00154-9 11165356

[B60] Stenberg G. (1994). Extraversion and the P300 in a visual classification task. Pers. Individ. Dif. 16, 543–560 10.1016/0191-8869(94)90182-1

[B61] Strobel A. Beauducel A. Debener S. Brocke B. (2001). Psychometrische und strukturelle Merkmale einer deutschsprachigen Version des BIS/BAS-Fragebogens von Carver und White. Z Diff. Diag. Psychol. 22, 216–227 10.1024//0170-1789.22.3.216

[B62] Wacker J. Chavanon M.-L. Leue A. Stemmler G. (2010). Trait BIS predicts alpha asymmetry and P300 in a go/nogo task. Eur. J. Pers. 24, 85–105

[B63] Walczyk J. J. Roper K. S. Seemann E. Humphrey A. M. (2003). Cognitive mechanisms underlying lying to questions: response time as a cue to deception. Appl. Cogn. Psychol. 17, 755–774 10.1002/acp.914

[B64] Wilkowski B. M. Robinson M. D. Troop-Gordon W. (2010). How does cognitive control reduce anger and aggression? The role of conflict monitoring and forgiveness processes. J. Pers. Soc. Psychol. 98, 830–840 10.1037/a0018962 20438227

[B65] Zuckerman M. DePaulo B. M. Rosenthal R. (1981). “Verbal and nonverbal communication of deception,” in Advances in Experimental Social Psychology, ed. Berkowitz L. (New York: Academic Press), 1–59

